# Learning curve of achieving competency in emergency endoscopy in upper gastrointestinal bleeding: how much experience is necessary?

**DOI:** 10.1136/bmjgast-2023-001281

**Published:** 2024-03-07

**Authors:** Gabriel Allo, Sonja Lang, Anna Martin, Martin Bürger, Xinlian Zhang, Seung-Hun Chon, Dirk Nierhoff, Ulrich Töx, Tobias Goeser, Philipp Kasper

**Affiliations:** 1 Department of Gastroenterology and Hepatology, Faculty of Medicine and University Hospital Cologne, University of Cologne, Cologne, Germany; 2 Division of Biostatistics and Bioinformatics, Herbert Wertheim School of Public Health and Human Longevity Science, University of California San Diego, La Jolla, California, USA; 3 Department of General, Visceral and Cancer and Transplant Surgery, University of Cologne, Cologne, Germany

**Keywords:** endoscopy, gastrointestinal bleeding, surgical training

## Abstract

**Objectives:**

The management of upper gastrointestinal bleeding (UGIB) has seen rapid advancements with revolutionising innovations. However, insufficient data exist on the necessary number of emergency endoscopies needed to achieve competency in haemostatic interventions.

**Design:**

We retrospectively analysed all oesophagogastroduodenoscopies with signs of recent haemorrhage performed between 2015 and 2022 at our university hospital. A learning curve was created by plotting the number of previously performed oesophagogastroduodenoscopies with signs of recent haemorrhage against the treatment failure rate, defined as failed haemostasis, rebleeding and necessary surgical or radiological intervention.

**Results:**

The study population included 787 cases with a median age of 66 years. Active bleeding was detected in 576 cases (73.2%). Treatment failure occurred in 225 (28.6%) cases. The learning curve showed a marked decline in treatment failure rates after nine oesophagogastroduodenoscopies had been performed by the respective endoscopists followed by a first plateau between 20 and 50 procedures. A second decline was observed after 51 emergency procedures followed by a second plateau. Endoscopists with experience of <10 emergency procedures had higher treatment failure rates compared with endoscopists with >51 emergency oesophagogastroduodenoscopies performed (p=0.039) or consultants (p=0.041).

**Conclusions:**

Our data suggest that a minimum number of 20 oesophagogastroduodenoscopies with signs of recent haemorrhage is necessary before endoscopists should be considered proficient to perform emergency procedures independently. Endoscopists might be considered as advanced-qualified experts in managing UGIB after a minimum of 50 haemostatic procedure performed. Implementing recommendations on minimum numbers of emergency endoscopies in education programmes of endoscopy trainees could improve their confidence and competency in managing acute UGIB.

**WHAT IS ALREADY KNOWN ON THIS TOPIC**

- Limited data suggest that patients with acute upper gastrointestinal bleeding (UGIB) have improved outcomes, if they receive endoscopy by more experienced endoscopists; however, it is unknown how many emergency procedures are necessary for an endoscopist to achieve competence in managing acute UGIB.

**WHAT THIS STUDY ADDS**

- Here for the first time, we describe a clear learning curve for endoscopists treating acute UGIB.
- Endoscopists with <10 oesophagogastroduodenoscopies with signs of haemorrhage had significantly higher treatment failure rates than experienced endoscopists or consultants.

**HOW THIS STUDY MIGHT AFFECT RESEARCH, PRACTICE OR POLICY**

- Implementing recommendations on minimum numbers of emergency endoscopies in education programmes of endoscopy trainees could improve their confidence and competency in managing UGIBs.

## Introduction

Acute upper gastrointestinal bleeding (UGIB) is a life-threatening event and remains a leading indication for emergency endoscopy. In recent years, the management of UGIB has seen rapid advancements with substantial innovations in triage, timing of endoscopy, medication, transfusion management and haemostatic procedures.^1–8^ Although research efforts to optimise patient care are advancing on all fronts, one crucial aspect of treatment has been almost completely neglected: the endoscopist.

While the benefits of the aforementioned innovations are evident, mastering the necessary skills to confidently and successfully apply these techniques requires intensive training and education to achieve profound understanding of bleeding pathophysiology, gastrointestinal anatomy and the intricacies of haemostatic devices. Besides accurate hands-on skills, endoscopists are constantly faced with challenges of accurate patient triage, initiation of medical and haemostatic procedures as well as real-time decision-making during life-threatening emergencies.

Considering these challenges, a profound education of gastroenterologists in the field of UGIB appears indispensable. However, recent analysis showed that only 60% of final year gastroenterology trainees from the UK expressed confidence in managing UGIB, with 82% desiring further training.^9^ The trainees reported a serious lack of exposure to haemostatic procedures and alarmingly, 18.6% of certified gastroenterologists had no exposure to any type of endotherapy.^10 11^ This alarming lack of trainee exposure to acute UGIB warrants a fundamental reassessment of current training programmes.^12^

It is well known from colonoscopy studies that the experience of the endoscopist and the number of successfully performed procedures improve the examination results.^13^ Similarly, establishing a minimum number of haemostatic procedures required before certification may significantly enhance trainees’ confidence and skill set. However, current guidelines lack specific recommendation in the context of UGIB.^14–16^

Therefore, this study aims to investigate how the endoscopists’ experience can impact outcomes in UGIB and to identify a minimum number of emergency procedures required to achieve competence in managing UGIB.

## Methods

We reviewed all oesophagogastroduodenoscopies (EGDs) which were performed at the University Hospital Cologne, between 1 January 2015 and 31 December 2022 because of suspected UGIB from our endoscopy documentation system. From this dataset, we identified cases with stigmata of recent haemorrhage (SRH), defined as spurting or oozing lesions, fresh blood in the upper gastrointestinal tract with or without detectable lesions, adherent clot as well as visible vessels as previously described.^17^ In case of variceal haemorrhage, active bleeding from varices, red whale or white nipple signs, a clot overlying the varices or oesophageal varices without any other obvious source of bleeding upper gastrointestinal tract were also defined as SRH. We included EGDs with adherent clots detected since the underlying bleeding sources demonstrated high rebleeding rates and guidelines recommend considering clot removal followed by endoscopic treatment of underlying high-risk stigmata.^14^ We hypothesised that increasing experience translates into higher rates of clot removal and endoscopic treatment resulting in lower rebleeding rates.

Cases were then included in our study analysis, if EGDs with SRH were performed by a member of our 24-hour on-call endoscopy team. Exclusion criteria were age <18 years, no follow-up 7 days after index EGD or initial presentation at another hospital.

During the study period, 18 physicians participated in emergency on-call duties. Seven were highly experienced consultants with >100 emergency procedures performed. Three of them had >5 years of experience in emergency endoscopies and four of them had >15 years of experience partly gained at prior employments at other hospitals. All analysed trainees started training at our tertiary centre. There were three trainees, who started endoscopy training within 3 years before the study period and eight trainees started endoscopy training during the study period. In our department, trainees start on-call duties after completing 4 years of general internal medicine and gastroenterology training and at least 6 months of supervised full-time endoscopy training. They are considered proficient for on-call duties by the discretion of the chief of our endoscopy unit when a proven independent ability to endoscopically treat bleeding in the context of EGD and colonoscopies is achieved. A virtual reality simulator or ex vivo animal models for training of both basic and advanced endoscopic techniques are not available at our centre.

Our on-call emergency endoscopy service is operating on a 365 days/24 hours basis from 17:00 hours to 08:00 hours on weekdays and 24 hours on weekends and holidays. On-call duties are performed by all endoscopists after they are considered sufficiently trained as described above. In addition, there is a backup team of three highly experienced consultants who can be called for on-demand supervision in case of unmanageable endoscopic emergencies during after-hours, weekends and holidays. In case of emergency bleedings and unstable patients, endoscopies are usually performed at the intensive care unit or emergency department with anaesthetic support or an experienced physician responsible for sedation.

To evaluate the endoscopic performance of each endoscopist, we chose the composite of treatment failure defined as unsuccessful primary endoscopic haemostasis (and necessary surgical or radiological intervention to achieve haemostasis) and/or rebleeding within 7 days after index EGD as the primary end point. Rebleeding was defined as new haematemesis or bloody nasogastric aspirate after initial successful endoscopic intervention, new melena or haematochezia after normalisation of stool colour or a drop of haemoglobin ≥20 g/L in the absence of an alternative explanation and SRH on repeated endoscopy. We did not include mortality in the composite end point since the majority of patients with UGIB die of non-bleeding-related causes, such as multiorgan failure, pulmonary conditions and terminal malignancies, which are not influenceable by the endoscopists’ procedural skills.^18^

For each EGD, we determined the number of EGDs with SRH ever performed by the respective endoscopist prior to the new-onset bleeding event by reviewing our endoscopy documentation system and defined this number as a surrogate marker for experience in emergency endoscopy. Supervision was defined as attendance of a more experienced endoscopist during endoscopy.

Descriptive analysis was conducted using Statistical Package for the Social Sciences, V.29 (IBM, Armonk, New York, USA) and R statistical software, V.3.5.1 (2018 the R Foundation for Statistical Computing). Categorical variables were presented as absolute and relative frequencies. Continuous variables were expressed as median and IQR. Univariate and multivariate analyses using the logistic regression model were performed to identify factors associated with treatment failure. Covariates were included in the multivariate analysis if their p value was <0.05 in the univariate analysis.

A learning curve was produced by plotting the number of prior EGDs with SRH performed by the respective endoscopist against the cumulative treatment failure rate until this level of experience. This approach gives a visual representation of the learning phase of endoscopists. We identified the coordinates with the most significant drop in treatment failure and thereby divided the study population into four groups. These groups were compared using one-way analysis of variance for parametric variables or Kruskall-Wallis test for non-parametric data, respectively. Additionally, we performed a moving average analysis by calculating the mean treatment failure rate for each EGD with SRH performed by the respective endoscopist over blocks of five endoscopies, mirroring the methods of a previously published study.^13^ A graph was created by plotting the mean treatment failure rate for all endoscopists against the number of EGDs with SRH performed. For categorical variables, χ^2^ test was used. Post hoc p values were obtained from Fisher’s exact test with adjustment for multiple comparisons using the false discovery rate. P values <0.05 were regarded as statistically significant.

## Results

**Figure 1:**
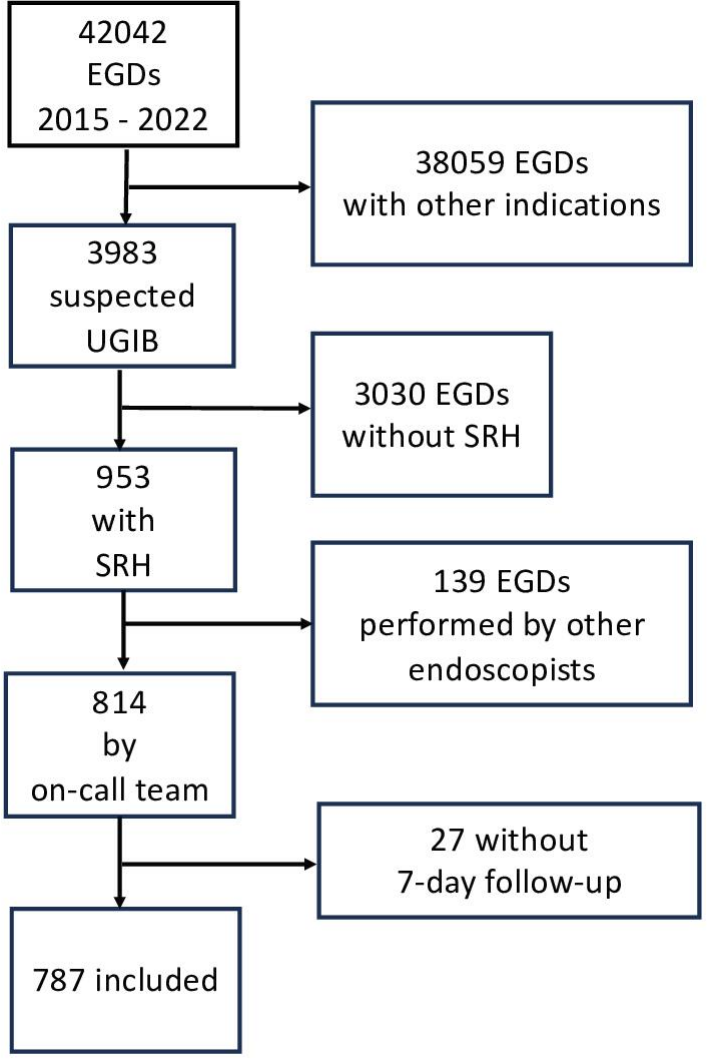
Study flow. EGD, oesophagogastroduodenoscopy; SRH, stigmata of recent haemorrhage; UGIB, upper gastrointestinal bleeding.

Flow chart of patient selection is shown in figure 1. Patients’ baseline characteristics are described in table 1. 787 cases were included in the final analysis of which 249 (31.6%) were women and the median age was 66 years. Outwith regular hours, 334 (42.4%) EGDs were performed and 108 (13.7) procedures were supervised. Duodenal as well as gastric ulcers were the most frequent bleeding source with 246 (31.3%) and 132 (16.8%) cases, respectively. Spurting and oozing bleeding was detected in 47 (6.0%) and 437 (55.5%) cases, respectively. Gastric and oesophageal varices were the source of bleeding in 116 (14.8%) cases and active bleeding from varices was detected in 84 EGDs (72.4% of gastric and oesophageal varices).

**Table 1:** Patient demographics, endoscopic findings and outcomes

	Total cohort
Age (IQR)	66 (19.0)
Women	249 (31.6)
History of bleeding	155 (19.7)
Mean number of packed red blood cell transfused (IQR)	2 (2.0)
Endoscopic experience (no. of previous EGDs with SRH) (IQR)	78 (70)
EGD during on-call duty	334 (42.4)
Supervision	108 (13.7)
Active bleeding	576 (73.2)
Bleeding source	
Gastric ulcer	132 (16.8)
Duodenal ulcer	246 (31.3)
Oesophageal varices	95 (12.1)
Gastric varices	21 (2.7)
Malignancy	55 (7.0)
Stigmata of recent haemorrhage	
Spurting	47 (6.0)
Oozing	437 (55.5)
Visible vessel	70 (8.9)
Adherent clot	117 (14.9)
Endoscopic therapy	
Metal clip	142 (18.0)
Injection therapy	18 (2.3)
Metal clip+injection	83 (10.5)
Band ligation	96 (12.2)
Argon plasma coagulation	94 (11.9)
Stent	1 (0.1)
Sengstaken-Blakemore tube	5 (0.6)
Haemostatic spray	24 (3.1)
Other	2 (0.2)
OTS clip	14 (1.8)
OTS clip+injection	6 (0.8)
Coagrasper	3 (0.3)
Haemostasis at the end of endoscopy	712 (90.5)
Radiology	48 (6.1)
Surgery	14 (1.8)
Rebleeding	146 (18.6)
In-hospital mortality	203 (25.8)

Numbers in brackets are percentages, if not stated otherwise.

EGD, oesophagogastroduodenoscopy; OTS, over-the-scope; SRH, stigmata of recent haemorrhage.

Endoscopic haemostatic interventions were performed in 643 EGDs (81.7%) and the most frequently applied procedures were haemoclips (297; 37.7%). Ongoing bleeding at the end of endoscopy was documented in 75 procedures (9.5%). Surgical or radiological interventions were necessary to achieve haemostasis in 14 (1.8%) and 48 (6.1%) of the patients, respectively. Treatment failure occurred in 225 (28.6%) cases while 203 (25.8) patients died during their hospital stay.

### Factors associated with treatment failure

In the underlying study, age over 65 years, a history of prior gastrointestinal bleeding, the need for more than two packed red blood cells, EGD during on-call duty, active bleeding detected during endoscopy, gastric ulcers, oesophageal varices, gastric varices as well as the experience level were significantly associated with treatment failure on univariate analysis. Multivariate analysis identified age over 65 years, the need for more than two packed red blood cells, EGD during on-call duty, active bleeding, gastric ulcers, oesophageal varices and the experience level as factors that are significantly associated with treatment failure (table 2).

**Table 2:** Factors associated with treatment failure

		cOR	95% CI	P value	aOR	95% CI	P value
Age (years)							
≤65	393 (49.9)	1.0					
>65	394 (50.1)	0.676	0.495 to 0.923	0.014	0.655	0.469 to 0.914	0.013
Sex							
Men	538 (68.4)	1.0					
Women	249 (31.6)	0.885	0.633 to 1.239	0.477			
History of bleeding							
No	632 (80.3)	1.0					
Yes	155 (19.7)	1.606	1.108 to 2.328	0.012	1.413	0.956 to 2.089	0.083
Number of packed red blood cell transfusions							
≤2	433 (55.2)	1.0					
>2	351 (44.8)	2.040	1.490 to 2.792	<0.001	1.859	1.331 to 2.596	<0.001
Time of EGD							
Working hours	453 (57.6)	1.0					
On-call duty	334 (42.4)	1.557	1.140 to 2.125	0.005	1.545	1.097 to 2.175	0.013
Supervision							
No	679 (86.3)	1.0					
Yes	108 (13.7)	1.060	0.679 to 1.655	0.797			
Stigmata of recent haemorrhage							
Visible vessel/adherent clot	211 (26.8)	1.0					
Active bleeding	576 (73.2)	2.023	1.375 to 2.976	<0.001	1.758	1.174 to 2.633	0.006
Bleeding source							
Gastric ulcers	132 (16.8)	0.532	0.334 to 0.847	0.007	0.515	0.315 to 0.840	0.008
Duodenal ulcers	246 (31.3)	1.353	0.976 to 1.877	0.069			
Oesophageal varices	95 (12.1)	0.394	0.218 to 0.711	0.001	0.325	0.175 to 0.604	<0.001
Gastric varices	21 (2.7)	2.837	1.188 to 6.778	0.014	1.661	0.664 to 4.154	0.278
Malignancy	55 (7.0)	1.348	0.756 to 2.404	0.311			
Experience							
<10	64 (8.1)	1.0					
10–50	219 (27.8)	0.718	0.403 to 1.278	0.26	0.638	0.348 to 1.171	0.147
51–100	152 (19.2)	0.52	0.279 to 0.969	0.039	0.459	0.237 to 0.889	0.021
>100	352 (44.7)	0.56	0.321 to 0.976	0.041	0.495	0.276 to 0.888	0.018

Numbers in brackets are percentages, if not stated otherwise.

aOR, adjusted OR; cOR, crude OR; EGD, oesophagogastroduodenoscopy.

### Learning curve

**Figure 2:**
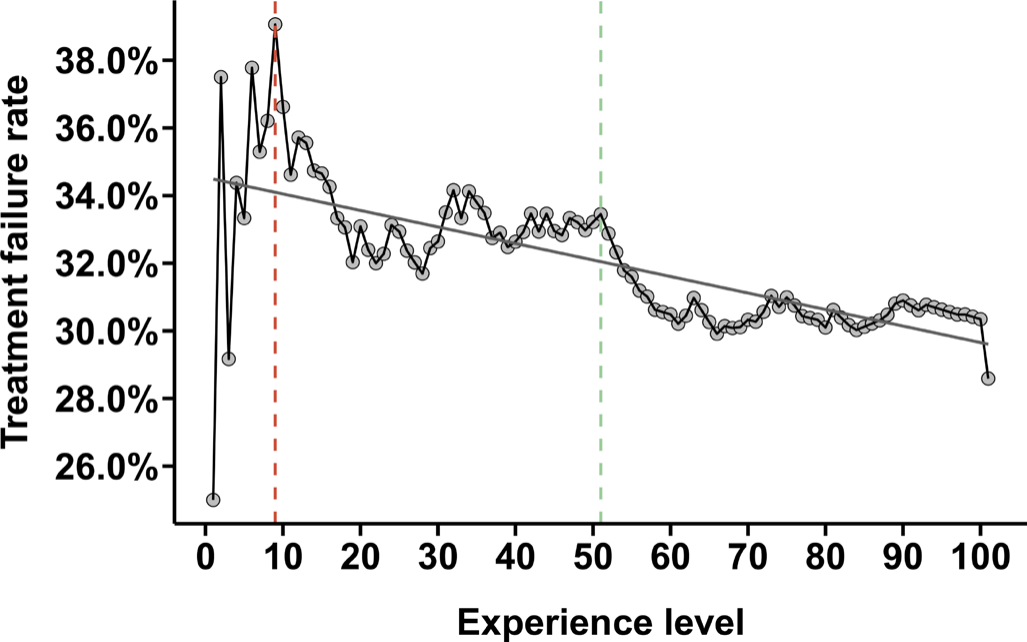
Endoscopists’ learning curve. Experience level=cumulative number of previous oesophagogastroduodenoscopies with stigmata of recent haemorrhage performed by endoscopists. The red line marks the point with the highest treatment failure rate followed by a steep drop. The green line marks the last point of the first plateau followed by a marked drop in treatment failure rates. The straight line displays the curve of best fit for the plot. Goodness of fit: R^2^=0.46, F-statistics=85.18, p<0.001.

Figure 2 displays the change of the cumulative treatment failure rate depending on the experience of the endoscopist. The curve shows a clear learning curve with a marked decline in endoscopic treatment failure rates after at least nine EGDs with SRH have been performed (39.1%). Furthermore, after reaching a first plateau phase between 20 and 50 EGDs performed, the learning curve demonstrates another decline after 51 EGDs (33.1%) followed by a second plateau phase up to 100 procedures. The moving average analysis also showed a clear decline in treatment failure rates with growing experience (online supplemental figure 1).

10.1136/bmjgast-2023-001281.supp1 Supplementary data

### Comparison of experience groups

**Figure 3:**
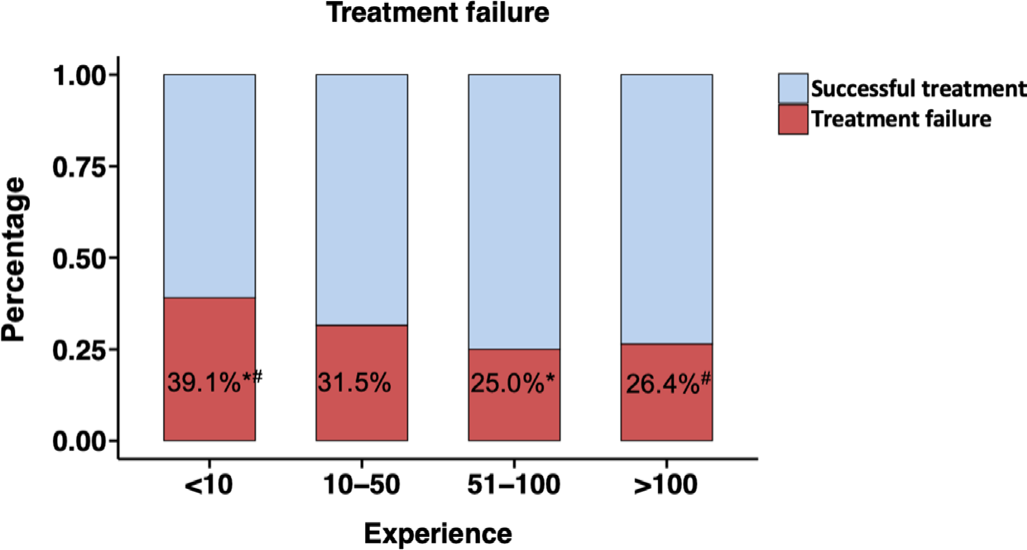
Treatment failure rates of experience groups. Experience=cumulative number of previous oesophagogastroduodenoscopies with stigmata of recent haemorrhage performed by endoscopists. Superscripted symbols indicate significant differences (*p=0.039; ^#^p=0.041). Treatment failure rate did not differ significantly between group <10 and 10–50 (p=0.26).

After demonstrating the association between the experience level and the treatment failure rate, we built experience level groups based on clear cuts on the learning curve. A total number of 64 (8.1%) emergency EGDs have been performed by endoscopists with an experience of <10 EGDs with SRH (low experience group) in the present study. 219 EGDs (27.8%) have been performed by endoscopist with an experience of 10–50 EGDs (intermediate experience group) and 152 (19.3%) by endoscopist with an experience of 51–100 EGDs (high experience group). 352 (44.7%) EGDs have been performed by experienced consultants with >100 EGDs of experience (consultants). After starting their endoscopy training, 5 of 11 trainees performed their first 50 EGDs with SRH within the study period and the median duration to reach this cut-off was 2.5 years (range 1.68–2.94 years). The treatment failure rates of the four groups were 39.1%, 31.5%, 25.0% and 26.4%, respectively (figure 3 and table 2). The group of endoscopists with low experience had a significantly higher treatment failure rate than the highly experienced group (p=0.039) and consultants (p=0.041), while the treatment failure rate did not differ significantly compared with the intermediate experienced group. Results were consistent in the multivariate analysis.

**Figure 4:**
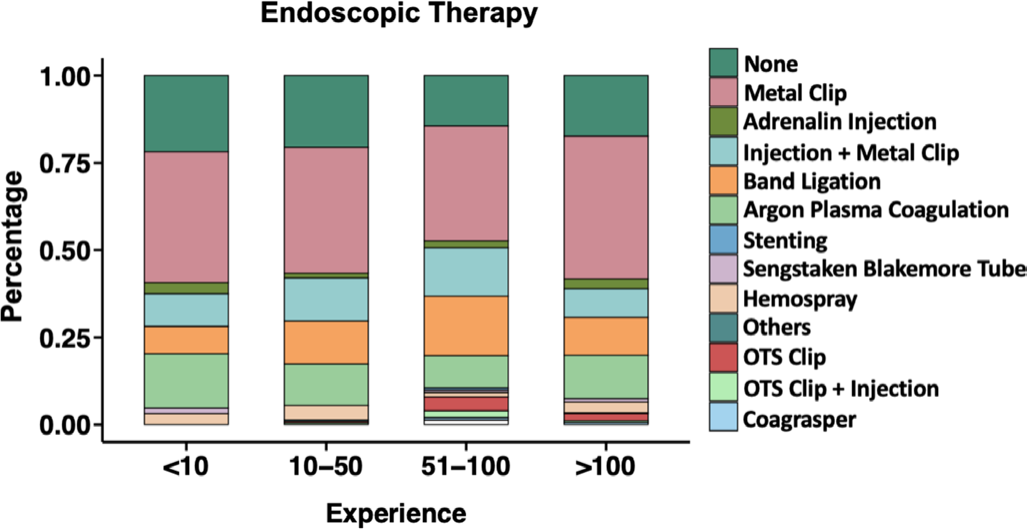
Endoscopic haemostatic interventions according to experience groups. Experience=cumulative number of previous oesophagogastroduodenoscopies with stigmata of recent haemorrhage performed by endoscopists. OTS, over-the-scope.

The high experience group performed emergency EGDs more frequently during on-call duty compared with the low experience group (p=0.002), the intermediate experience group (p=0.012) and the consultants (p=0.01). The low experience group performed their emergency EGDs more frequently under supervision compared with the intermediate experience group (p=0.003), the high experience group (p<0.001) and the consultants (p<0.001) (online supplemental table 1). Interestingly, there was no significant difference in the choice of endoscopic treatment methods. Although innovative haemostatic devices such as haemostatic spray and over-the-scope clips (OTSCs) were used more frequently by more experienced endoscopists compared with the low experience group without reaching statistical significance (figure 4 and online supplemental table 1). Furthermore, in a subgroup analysis of all ulcers with adherent clots, the low experience group received endoscopic haemostatic therapy in only 9.1%, differing significantly to 41.9%, 58.3% and 37.3% in the intermediate experience group, the high experience group and the consultants (p=0.049), respectively (data not shown).

10.1136/bmjgast-2023-001281.supp2 Supplementary data

## Discussion

In this retrospective study, we found that the experience of endoscopists was a significant factor for a successful treatment in acute UGIB. The treatment failure rate declined with increasing experience and endoscopists with experience of <10 EGDs with SRH performed had a higher treatment failure rate compared with those with >51 procedures.

The importance of the endoscopists’ experience in managing UGIB has not been extensively studied, and due to missing data, most guidelines on the management of UGIB do not recommend a specific number of emergency EGDs before considering an endoscopist as competent to perform emergency procedures independently and without supervision.^14 15^ To date, there have been only few studies examining the role of the endoscopists’ experience on the success of the haemostatic emergency procedures.

An Italian study from 2005 compared the performance of ‘experienced’ and ‘less experienced’ endoscopists in managing non-variceal UGIB. Seven endoscopists were divided into two groups based on the number of haemostatic endoscopic procedures performed before the study. Patients with EGDs performed by endoscopists with >100 emergency procedures had lower rebleeding rates and transfusion requirements than patients treated by endoscopists with 40–70 emergency procedures.^19^ The authors suggest that the lower appropriateness of applied endoscopic treatment by less experienced endoscopists might have led to higher rebleeding rates, particularly in cases of Forrest IIb ulcers, where the less experienced endoscopists were often not following guidelines, fearing uncontrollable bleeding after recommended clot removal.

Our results align with the above-mentioned study, as we also found an association of lower experience and higher treatment failure rates. However, our study findings extend beyond the simple comparison of young and highly experienced endoscopists by revealing a clear learning curve. The given curve indicates an initial learning phase of about 9 EGDs with SRH, followed by a phase of proficiency until 51 procedures and thereafter, a third phase of competence. The risk factors associated with treatment failure identified in our study and patient characteristics did not differ between experience groups except that group 51–100 performed EGDs more frequently during on-call service. Interestingly, there was a trend towards more experienced endoscopists using innovative haemostatic procedures such as OTSCs or haemostatic spray more frequently. This observation of choosing more specific haemostatic devices might have impacted outcomes, as recent studies showed a significant benefit of OTSCs in high-risk lesions and in case of rebleeding compared with standard endoscopic therapy.^4 20 21^ Additionally, the results of recent studies indicate that the use of haemostatic spray might result in more favourable outcomes in gastrointestinal tumour bleeding, which is associated with striking rebleeding and mortality rates.^22 23^ However, it is likely that appropriate training would increase confidence in applying these modern techniques and improve a patient’s outcome.

Similar to the aforementioned Italian study,^19^ endoscopists with lower experience also did not remove adherent clots in the vast majority of cases in the present study, likely due to fear of subsequent uncontrollable bleeding after clot removal. This underlines the importance of sufficient training to establish proficiency and confidence in all available endoscopic haemostatic techniques as well as in complication management.

Recent surveys from the UK displayed a lack of confidence among gastroenterology trainees in managing UGIB. Here, only about 60% of gastroenterology trainees expressed confidence in managing UGIB in their final year and 82% desired further training in haemostatic procedures.^9^ When analysing the portfolios of certified gastroenterologist in the UK, 18.6% had no exposure to any type of endotherapy, while only 37.1% performed band ligation and 50.7% placed a metal clip.^10^ Furthermore, a significant decline of trainee exposure to acute UGIB from 76% to 15% was observed in the UK, with about one-quarter of trainees doubting to become confident until the end of specialty training.^12^

These alarming results further highlight the need for comprehensive training programmes, where trainees are able to perform challenging UGIB cases under supervision. Several training initiatives have been postulated in recent years, such as individual endoscopy lists and tailored training programmes, which aim to increase trainees’ exposure to UGIB without risking patient safety.^24^ However, although recommendations exist, these are not sufficiently implemented and applied in everyday clinical practice.

Our study results support the idea of a requirements catalogue for endoscopic examinations during the endoscopy training programme, including minimum numbers of endoscopic emergency procedures (eg, haemostatic interventions) required before training is regarded sufficient, since our learning curve revealed a clear association of experience in haemostatic procedures and patient outcome. Training methods to improve trainees’ exposure to acute UGIB in real-life setting might be the implementation of protected and supervised morning slots for stable overnight patients with UGIB. Besides collecting experience during regular practice, implementing gastrointestinal haemostatic training courses in the regular education programme can significantly improve endoscopists’ skills and confidence in managing emergency situation.^25^ However, participants should perform hands-on-training to improve their skills, because mere knowledge-based training had no effect on the endoscopists’ performance.^26^ Since patient-based training can cause discomfort or even harm patients, simulation-based training modalities such as ex vivo animal models or computerised virtual reality simulators are commonly used to improve trainees skills in a safe environment and represent promising approaches to train basic and advanced endoscopic procedures.^27 28^

Since our learning curve shows a first plateau of treatment failure rates after about 20 EGDs with SRH were performed, we recommend this as a minimum for an endoscopists to be considered proficient to perform emergency procedures independently. Endoscopist might be considered highly qualified experts in managing UGIB after a minimum of 50 haemostatic procedure performed. Our results also imply that hospitals should focus on maintaining a sufficiently trained on-call endoscopy service team to improve clinical management of patients with UGIB.

Initially, the treatment failure rates of 28.6% in our study might appear high, however, the rebleeding rate was not higher than in other studies on endoscopic high-risk stigmata.^29^ Furthermore, the composite primary end point also included need for surgical and radiological haemostatic intervention as well as ongoing bleeding at the end of endoscopy, which occur naturally more frequently in cases with high-risk stigmata than in unselected study populations with UGIB.

Compared with other studies on non-variceal UGIB, mortality rates were higher in our study. This might be explained by the significant number of cases with in-hospital bleeding, variceal bleeding and bleeding from malignancies included in this study population, which are associated with high mortality rates of up to 20%, 32% and 47.5%, respectively.^22 30 31^

Despite higher treatment failure rates of endoscopists with low experience, the mortality rates tended to be lower in this group. However, these results have to be interpreted with caution, since endoscopists with low experience performed their endoscopies more frequently during regular hours under supervision. Similar to our study, a meta-analysis identified admission during off-hours as a risk factor for unfavourable outcome^32^ and the more favourable circumstances during regular hours might have improved patient’s survival. Moreover, urgent endoscopy within 6 hours is not recommended for stable patients with UGIB^1 33^ and thereby stable patients receive endoscopy more frequently during regular hours compared with critically ill and instable patients, who require urgent endoscopy directly after haemodynamic stabilisation.^14 15^

The limitations of our study derive from its retrospective and monocentric design. Since this study was conducted at a tertiary centre with 24 hours/day endoscopy service with an experienced consultant as an on-demand back-up, the transferability of our results might be limited towards other institutions with less experience and resources. Additionally, due to the retrospective design, we were unable to clarify to which extent a supervising endoscopist supported the main endoscopist during the procedure. Furthermore, we were unable to retrospectively obtain the exact timing of endoscopy after presentation of bleeding. Therefore, we were not able to analyse the impact of experience level on timing of endoscopy and its influence on patients’ outcomes.

In conclusion, our results demonstrate a clear learning curve in endoscopists’ performance in acute UGIB management. Treatment failure rates were significantly higher during the early learning phase. Based on our data, we recommend a minimum of 20 EGDs with SRH for an endoscopists to be considered proficient to perform emergency endoscopy independently. Endoscopist might be considered highly qualified experts in managing UGIB after a minimum of 50 haemostatic procedure performed. Implementing individual endoscopy lists in the education of trainees could improve their competence in managing acute UGIB.

## Data Availability

Data are available on reasonable request.
