# Supplementary figures and images for: Learning curve of achieving competency in emergency endoscopy in upper gastrointestinal bleeding: how much experience is necessary?
Source: BMJ Open Gastroenterol. 2024 Mar 7;11(1):e001281. doi: 10.1136/bmjgast-2023-001281 (PMC10921515; doi:10.1136/bmjgast-2023-001281)

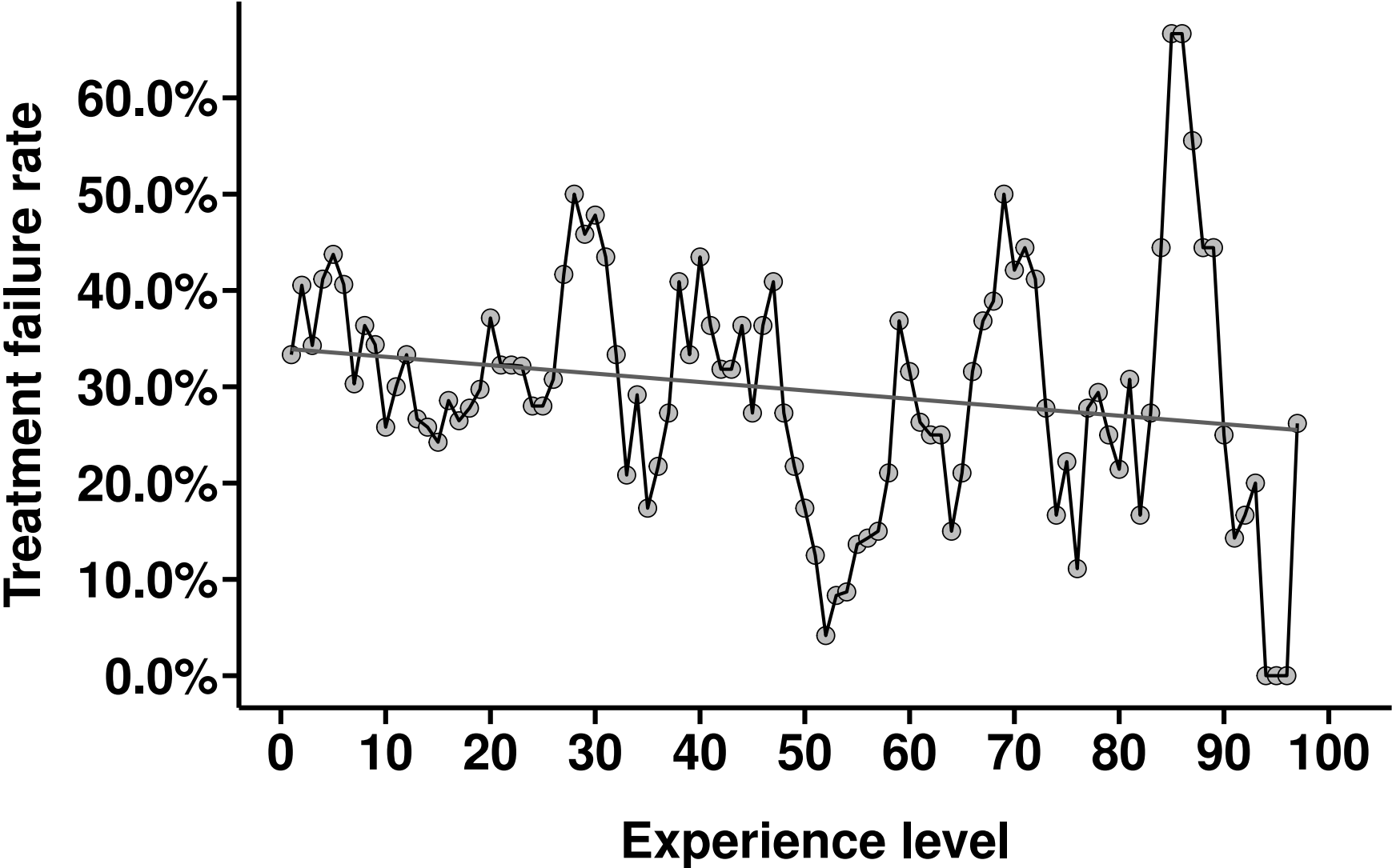

Supplement: Supplementary data [file bmjgast-2023-001281supp001.pdf]
