# Supplementary material for: Learning curve of achieving competency in emergency endoscopy in upper gastrointestinal bleeding: how much experience is necessary?
Source: BMJ Open Gastroenterol. 2024 Mar 7;11(1):e001281. doi: 10.1136/bmjgast-2023-001281 (PMC10921515; doi:10.1136/bmjgast-2023-001281)
Supplement: Supplementary data [file bmjgast-2023-001281supp002.pdf]

**Supplementary Table 1.** Comparison of patients' baseline characteristics, endoscopic findings and outcomes according to experience group

| | **<10** | **10-50** | **51-100** | **>100** | **p** |
|---|---|---|---|---|---|
| Age, median (IQR) | 68 (17.2) | 65 (18.0) | 64 (18.0) | 67 (19.0) | 0.340 |
| History of bleeding | 12 (18.8) | 42 (19.2) | 23 (15.1) | 78 (22.2) | 0.331 |
| EGD during on-call duty | 18 (28.1) | 89 (40.6) | 84 (55.3) | 143 (40.6) | 0.001 |
| Blood transfusions, median (IQR) | 2.0 (3.2) | 2.0 (2.0) | 2.0 (2.8) | 2.0 (2.0) | 0.480 |
| Supervision | 46 (71.9) | 43 (19.6) | 13 (8.6) | 6 (1.7) | <0.001 |
| Active bleeding | 15 (23.4) | 56 (25.6) | 42 (27.6) | 98 (27.8) | 0.853 |
| Source of bleeding | | | | | 0.798 |
| Gastric ulcers | 9 (14.1) | 33 (15.1) | 22 (14.5) | 68 (19.3) | |
| Duodenal ulcers | 22 (34.4) | 72 (32.9) | 47 (30.9) | 105 (29.8) | |
| Esophageal ulcers | 2 (3.1) | 7 (3.2) | 4 (2.6) | 16 (4.5) | |
| Mallory-Weiss syndrome | 2 (3.1) | 4 (1.8) | 3 (2.0) | 3 (0.9) | |
| Gastritis | 1 (1.6) | 5 (2.3) | 0 (0.0) | 10 (2.8) | |
| Portal hypertensive gastropathy | 1 (1.6) | 3 (1.4) | 2 (1.3) | 10 (2.8) | |
| Esophageal varices | 6 (9.4) | 25 (11.4) | 27 (17.8) | 37 (10.5) | |
| Gastric varices | 1 (1.6) | 8 (3.7) | 6 (3.9) | 6 (1.7) | |
| Malignancies | 6 (9.4) | 17 (7.8) | 10 (6.6) | 22 (6.2) | |
| Other bleeding sources | 12 (18.8) | 36 (16.4) | 24 (15.8) | 59 (16.8) | |
| Bleeding source not identifiable | 2 (3.1) | 9 (4.1) | 7 (4.6) | 16 (4.5) | |
| Stigmata of recent hemorrhage | | | | | 0.697 |

| | | | | | |
|---|---|---|---|---|---|
| Spurting | 5 (8.6) | 14 (7.6) | 12 (9.9) | 16 (5.2) | |
| Oozing | 40 (69.0) | 118 (64.1) | 73 (60.3) | 206 (66.9) | |
| Visible vessel | 2 (3.4) | 21 (11.4) | 12 (9.9) | 35 (11.4) | |
| Adherent clot | 11 (19.0) | 31 (16.8) | 24 (19.8) | 51 (16.5) | |
| Endoscopic Therapy | | | | | 0.317 |
| None | 14 (21.9) | 45 (20.5) | 22 (14.7) | 61 (17.3) | |
| Metal Clip | 24 (37.5) | 79 (36.1) | 50 (33.3) | 144 (40.9) | |
| Adrenalin Injection | 2 (3.1) | 3 (1.4) | 3 (2.0) | 10 (2.8) | |
| Metal Clip + Injection | 6 (9.4) | 27 (12.3) | 21 (14.0) | 29 (8.2) | |
| Band Ligation | 5 (7.8) | 27 (12.3) | 26 (17.3) | 38 (10.8) | |
| Argon Plasma Coagulation | 10 (15.6) | 26 (11.9) | 14 (9.3) | 44 (12.5) | |
| Stenting | 0 (0.0) | 0 (0.0) | 1 (0.7) | 0 (0.0) | |
| Sengstaken Blakmore Tube | 1 (1.6) | 0 (0.0) | 1 (0.7) | 3 (0.9) | |
| Hemospray | 2 (3.1) | 9 (4.1) | 2 (1.3) | 11 (3.1) | |
| Other | 0 (0.0) | 1 (0.5) | 0 (0.0) | 1 (0.3) | |
| OTS-Clip | 0 (0.0) | 1 (0.5) | 6 (4.0) | 7 (2.0) | |
| OTS-Clip + Injection | 0 (0.0) | 1 (0.5) | 3 (2.0) | 2 (0.6) | |
| Coagrasper | 0 (0.0) | 0 (0.0) | 1 (0.7) | 2 (0.6) | |
| Hemostasis at the end of endoscopy | 56 (87.5) | 192 (87.7) | 136 (89.5) | 328 (93.2) | 0.121 |
| Surgery | 2 (3.1) | 4 (1.8) | 4 (2.6) | 4 (1.1) | 0.546 |
| Radiology | 3 (4.7) | 18 (8.2) | 12 (7.9) | 15 (4.3) | 0.181 |
| In-hospital Mortality | 8 (12.5) | 59 (27.2) | 41 (27.0) | 95 (27.0) | 0.090 |
